# An Account of Some Rare Cases of Disease

**Published:** 1849-06

**Authors:** W. L. Sutton

**Affiliations:** Georgetown, Ky.


Art. IV.—*An Account of some Rare Cases of Disease.* By W. L. Sutton, M.D., of Georgetown, Ky.

As the cases herein detailed, excited in me much interest at the time, and as they afford some matter for reflection, I think proper to lay them before the profession.

Some time in June last, Meacham Webb, of this vicinity, lost a cow by some disease which excited little attention, except as to its sudden occurrence. The cow was skinned and hauled out into a clover field, in which was a lot of hogs. Within a few days he lost thirty of his hogs. Still no importance was attached to the circumstance, because he "knew that carcases would frequently kill hogs in hot weather." Some weeks now intervened, during which the cattle seemed healthy; they then commenced dying with considerable rapidity. It was now observed that one would quit chewing her cud; stand drooping; presently would appear a slight swelling of the throat; trifling running from the mouth and nose; thick, heavy breathing, drawing up of the abdomen, and shrinking of the body. The respiration would become more and more laborious until death, which occurred in from ten to thirty-six hours from the time when the first symptoms were observed. Convulsions invariably preceded death, and the body soon became very much swollen. A yoke of oxen was worked until noon, when one of them was observed to be unwell. It was turned out, and died in about two hours. A cow would be milked at night, being to all appearance entirely healthy, and found dead next morning. No animal after it was observed to be unwell, recovered.

During the prevalence of the epizootic, the cattle were changed into different pastures, in some of which there was pond water, in others running water, without benefit. The cattle were not fed at all, the pastures being fine.

The early part of summer was dry, but the latter very wet. The last of the cattle died about the 12th of September, and a horse on the 17th, affected very much in the same way. In all, twelve cattle and two horses died. All the cattle were skinned, and, after the first, burnt. But three were examined internally, two by the negroes in presence of Mr. Ford, and one in the presence of Mr. Webb. Mr. Ford reported the spleen to be much softened. Mr. Webb did not observe any appreciable lesion in the spleen or any other organ. If there had been any malignant pustule on the cattle, it could scarcely have escaped detection while they were being skinned.

On the 15th September, William, a negro man belonging to Mr. Webb, and who had assisted in skinning all the cattle, came to my office with an ulcer on the outer part of the forearm near the elbow, something larger than a quarter of a dollar, in which was an eschar black in the center, having a diameter something more than half that of the ulcer, the circumference of a dirty white. The sound skin came up to the ulcer in a regular circle, edges smooth and round; the skin being separated from the subjacent tissue at different points of its circumference, from a half to one and a half inches. Between the eschar and the skin was discharged a considerable quantity of ichorous fluid, through which bubbles of air frequently arose. The arm below the elbow was very much swollen, having a hard brawny feel, not very painful. He informed me that his arm had been much more swollen up to the elbow, and painful—that about two weeks before, he had been quite sick; at which time, a small blister was observed at the point now occupied by the ulcer. I also subsequently learned that Dr. Desha had seen him at the time, bled him freely, and gave him a dose of calomel, which had afforded him very decided relief. He had also directed the blister to be covered with calomel, and the arm to be poulticed. Reflecting that the swelling of the arm was said to have subsided considerably, I thought it altogether likely that the arm would go on to get well without any material change of treatment; yet I deemed it proper to advise it to be cauterised. The master concluded to defer it, at least until the next day.

On the 16th, the arm presented very much the same appearance, except that the surface of the ulcer was thought to be somewhat larger, and instead of ichor, it was freely discharging dissolved blood, through which particles of air still escaped. The eschar was separated with the knife, which left a space that would have received the large end of a hen's egg to about the middle. A circular bar of iron, corresponding in size to the ulcer, was heated to a white heat and applied in the presence of Drs. Paxton, Barclay, and J. Sutton, some of whom thought that from the depth to which the iron went, the bone must have been burned. Such, however, was not the case. We intended to incise the skin from the edge of the ulcer at right angles, so as to admit applications between the skin and cellular membrane as far as they were separated. In making the first, it was carried a little farther than necessary, and a vein was cut which bled very freely. Being not without fear of bleeding in such cases, I instantly put my finger upon it and restrained the hemorrhage until the cavity was filled with Peruvian bark, and a bandage applied up to the elbow. Nothing unusual or unpleasant occurred afterwards; the swelling subsided; and the sore healed as soon as could reasonably have been desired.

On the evening of the same day upon which William was cauterized, I was desired to visit the two children of R. F. Ford, Esq., son-in-law to Mr. Webb, who had had his residence at Mr. W.'s house, during nearly the whole time the cattle were suffering. Of this fact, I was not aware for nearly a week after my first visit.

Benjamin, the older boy, had been taken on the 9th with what was judged by Dr. Desha to be measles. There was said, however, to have been no hoarseness, sneezing, or redness of eyes, and but little cough. On the 14th, some swelling appeared about the angle of the left lower jaw, which increased in size rather rapidly. The child had always been delicate and leucophlegmatic in temperament; and at the time of my visit had some fever, which his father represented as having been uninterrupted from the beginning. The cutaneous eruption had disappeared. The tongue was neither red nor dry, but presented the appearance of one in a case of mild scarlet fever considerably advanced in convalescence: rather smooth, the velvety appearance gone, papillæ enlarged. Very considerable swelling at the angle of the jaw, presenting the appearance of those which occasionally occur towards the close of bad cases of scarlet fever, bilious fever, dysentery, etc., which are frequently ascribed to the too free use of mercury. This child had taken no medicine of any kind. Wishing if possible to prevent suppuration, I applied a blister on it immediately. By the 19th the swelling had increased considerably, and on the part then most prominent, which however, had not been covered by the blister, was a darkish spot. Here was a collection of ill-looking thin pus under the cuticle; it was opened, and this with a small quantity of good looking pus was discharged, altogether not exceeding a tablespoonful: Sloughing immediately commenced and progressed steadily but slowly till the 23d when he died. For two days before death there had been considerable cerebral symptoms. He also had considerable diarrhea, and for twelve hours occasional vomiting of grass green matter.

Meacham, the younger child, two years old, at my visit on the 16th, had considerable fever; pulse quick; respiration between fifty and sixty; skin hot and dry; on the abdomen were a few red spots not elevated. His tongue presented the same appearance as Benjamin's. He had, upon the upper part of the right thigh, a considerable swelling, which extended from the fold between the abdomen and thigh nearly to the knee. There was nothing in its appearance to distinguish it from that inflammation which precedes an ordinary large abscess. This swelling had been observed on the 12th, and it was said had progressed very slowly. Under the hope that this might be discussed, it was directed to be rubbed with volatile liniment containing laudanum, and hot flannels applied frequently. No improvement having taken place, on the 18th the thigh was directed to be poulticed to promote suppuration.

19th. Upon removing the poultice a small depression half the size of a split pea was observed, as if some hard substance had been interposed between the poultice and thigh. There was now a slight tinge of the blue observable on the surface of the swelling.

On the 20th, the small depression, before mentioned, had ulcerated. This was immediately over the femoral artery at its exit from the groin. This ulceration progressed steadily but slowly until death, which took place on the 4th of October.

Poultices of oak bark, of Peruvian bark, lotions of kreosote, of laudanum; applications of castile soap, producing no impression, lunar caustic was applied, followed by bandage from the toes up, on the 24th of September. The bandage and accompanying dressings appeared to exert a beneficial influence upon the swelling of the thigh—the swelling diminished somewhat, the hardness abated at the surface, but it was very evidently deeper; the skin improved considerably in color and appearance, but as before mentioned, the ulcerated surface continued to enlarge, and never afforded a decidedly healthy looking pus except on the morning of the 1st October. As a drawback upon the benefit resulting from the use of the bandage, that portion of the abdominal surface which was below the turn of the bandage around the body, on the right side, began to swell. We attempted to remedy this by applying pressure over all this swelled part. In this we failed, as I think, because a slight bending of the thigh upon the body would remove the degree of pressure first applied. The scrotum and penis became much swelled, and ulceration set up above

Poupart's ligament. Under this state of things, I felt myself constrained to remove the pressure from the abdomen, preferring no pressure to that which acted unequally. For several of the last days of life, erysipelatous inflammation of a migratory, evanescent character appeared upon the hips, back, and knee.

On the 2d October, the powers of life seemed to give way, and ulceration progressed very rapidly, so that at death, the sheath containing the femoral vessels and nerve was exposed for an inch and a half. In this case I had the benefit of the advice of Drs. Craig and Winston.

It will be observed that I have said nothing as to the general treatment. Of course that was not neglected; but my present business lies more particularly with the local affection. Suffice it to say, that the general health seemed to improve considerably; the fever, with attendant quickness of pulse and respiration, subsided, and for several days before the 2d of October, we were encouraged to hope that the general improvement of health, and of the appearance of the thigh would eventually check the ulcerative process.

Henrietta, a black girl, nurse to Meacham, on the 16th Sept., had a tongue entirely similar to that of the two children. She also seemed bloated, as is frequently the case after scarlet fever. She had been complaining longer than the children, and was supposed to have had the same disease; swelling of the feet and face, however, had been among her earliest symptoms. She had never been entirely confined, and was thought to be improving until the 19th of September, when, without any assignable cause, she commenced trembling, had an impression that she was about to die, and talked rather sensibly upon that subject for a negro of her age. She had several paroxysms of trembling, some of which approached closely to convulsions. I found her at my visit apparently asleep, very much as is frequently the case after an epileptic fit. After she came to her senses, she had no knowledge of what had passed, but complained of some pain in the thorax, had some cough, and indeed seemed to have a slight attack of pneumonia, from which she slowly recovered.

David, aged four years, was complaining on the 22d of September; had the same kind of tongue as the three preceding cases, some tumefaction of the abdomen and swelling of the feet, slight cough. Under the impression that he had worms, I directed him to have a mercurial, to be followed next morning by a vermifuge.

23d. Was thought to be better; did not see him; went to bed lively, but was discovered about midnight to be stupid. I saw him at 2 o'clock, A.M. of 24th; entirely insensible; pulse 180, as near as I could determine; belly distended. Had taken a dose of calomel before my arrival. Supposing that he had eaten something improper, gave him, in different portions, about two drachms of ipecac., which produced no effect. He subsequently took about a scruple of calomel, and had blisters to the neck, epigastrium, ancles, and wrists. During the day he had several discharges from his bowels of bright spinach color. In this and the previous day he passed a few lumbrici. He died at 4 P.M., on the 25th.

Twenty hours after death examined the body in presence of Drs. Winston, Craig, Paxton, and J. Sutton. In turning down the scalp, there was an extravasation of blood near the sagittal suture as if he had been lately stricken there. There was no fracture and no injury corresponding thereto on the inside of the cranium. The superficial veins of the brain were loaded with blood, but the brain did not seem to be the seat of inflammation. Very little water, if any, in the ventricles.

*Thorax.*—Heart and lungs healthy.

*Abdomen.*—The stomach, liver, spleen, and bowels all appeared externally healthy, but upon opening the stomach, almost the whole internal surface was found highly inflamed. The gall-bladder was also much distended with spinach colored bile.

I presume there will be little doubt that the case of William was one of malignant pustule. It will be observed that bleeding and purging, so much dreaded on the continent of Europe, were followed by marked relief. The man expressed in the strongest terms his sense of amendment after the application of the cautery, and the healing went on as rapidly as could be expected for a wound of that depth.

A grave question presents itself as to the other cases detailed above. Were they, or any of them connected with the diseased cattle? All the persons had been living on the farm nearly the whole time of the epizootic. We know that the flesh of diseased cattle is capable of producing the malignant pustule, and perhaps other grave affections. But no flesh from either of these animals had been eaten save by the hogs. Is the milk of a diseased cow capable of communicating the affection? I do not know of any authority upon this point. If these cases were at all connected with the epizootic it must have been through the milk. During the time three milch cows died, but no one was milked after it was known to be diseased. Indeed I am of opinion that none of them were known to be diseased until they were found dead. Add to this, Benjamin, the older child, was in the habit of eating but little milk. It will be remembered that Dr. Desha considered the two children as affected with measles. The swellings, too, had nothing to distinguish them from those of ordinary abscesses, before sloughing commenced; and they had nothing of the appearance peculiar to malignant pustule.

On the other hand, it was a little singular that two children should be affected at the same time with affections so very similar in their course—that course being altogether uncommon, without a community of origin. Was there a mistake in the diagnosis? I have full confidence in Dr. Desha's judgment, yet am I not without misgivings that in this instance it may have been incorrect. In the first place, the origin of these cases was supposed to have been connected with that of an Irshman's child in the neighborhood. This child I saw on the 26th of August and 5th of September. She died on the 7th or 8th. She was said to have had an eruption, but I did not see it; and from the description given did not suppose it to have been either measles or scarlatina. When I saw her last, she had a swelling at the angle of the left lower jaw, which seemed to have nothing peculiar in it, and was said by the father to be perfectly similar to one which she had had some year before. At death this had neither suppurated nor ulcerated.

Again, there was said to have been no cough, hoarseness, or watery eyes, so characteristic of measles. And again, an eruption appeared on Meacham's abdomen in the latter part of his illness, which the family recognized as the same which existed in the beginning. It is not usual to have swellings at the angles of the jaws in measles. I do not remember ever to have seen one; though I certainly know no reason why they might not follow bad cases of measles, as well as those of other forms of fever. But these risings appeared too soon to be the consequence of the fevers—one on the third, the other on the fifth day of illness. There is another consideration not without some weight: there were no other cases of measles in the neighborhood. This could scarcely have been the case had these been measles, as Mr. Webb had a large family of black children, through one of whom the infection must have been received, if at all. It is right to state, that that one was Henrietta, the subject of the fourth case. To conclude, there was no desquamation of the cuticle, except on the leg which was bandaged.

But say that the children were affected with scarlet fever? Then all the objections above mentioned, except as to the swelling at the angle of the jaw, lie with equal, some of them with more, force. The tongue of each one of the four had the same appearance, and no one of them exhibited the smooth, red, glossy appearance which marks a severe case of scarlet fever—such as would be apt to give rise to glandular swellings. The edematous condition of Henrietta was observed not only when I saw her, at which time it might have been ascribed to scarlatina, although it was full early, but also at the very commencement of her indisposition.

In each of the four cases, there was a slight cough, (in Henrietta's case this was observed before the pneumonic symptoms came on); a perfect similarity of tongue —such as is not observed ordinarily, in the midst of a severe attack of any fever. This state of tongue continued throughout the life of Benjamin, to the last days of Meacham; during the course of Henrietta's attack; and as long as it was to be observed in the negro boy. It was one indicative of intestinal irritation. Accordingly we find in the negro boy, the passing of green stools of greater intensity than those under the use of calomel in cerebral disease; and after death, the gall-bladder distended with bile of the same color; and in addition the mucous coat of the stomach highly inflamed. In Benjamin, the vomiting of the same colored matter in the last days of his life. Meacham also passed green stools in the last days, but his were not of a character so exalted.

Nor must we forget the fact, that although in David the stomach was highly and extensively inflamed, there was not only no spontaneous vomiting, but two drachms of ipecacuanha failed to bring it on. Could this have been the case if the inflammation had been of the simple ordinary kind?

I have said that the ulceration in Benjamin and Meacham had not the characters of malignant pustule, yet I do not know any affection to which they bore a greater analogy than to that; and Dr. Craig very justly remarked that, "although it certainly was not carbuncular inflammation, it looked very much like it."

It may not be irrelevant to state that Mr. Ford is very strongly persuaded that all these cases, of man and beast, were the result of poisoning. He lost four pointer dogs, as he supposes, from the exhibition of strychnine, which in their mode of death resembled the cattle very much; but did not swell their bodies after death.

There were circumstances of a very suspicious character in reference to some negroes of the family; but from the history of the epizootic and of the diseases of the children, we were not disposed to favor his opinion. At the time we examined David we were not aware of any suspicion of foul play respecting him.

*November* 2.—After I had written this accout to the last two paragraphs, Mr. Ford brought in the fourth dog for examination, giving the following history. "Yesterday I was out hunting nearly all day, the dog entirely well. Upon my return at night, I directed the dog to be fed. It was done in my presence with meat from the table. Some little time after, I went out of the house, the dog went with me and returned with me, but was out of my sight some ten minutes. Upon returning into the house the dog laid down upon the mat on which he usually slept. Next morning he was found dead, apparently without having moved. He and some others remained in the room until late bedtime, himself all night, no notice having been attracted to the dog.

*Autopsy.*—The muscles very rigid; the jaws firmly clenched, so as to embrace the tongue between the teeth. *Cranium.*—Some effusion on the surface of the dura mater; the veins on the surface of the brain engorged; a coagulum in the left mastoid cell; blood fluid in the veins. *Thorax.*—Lungs red and engorged with blood. *Heart.*—Auricles on both sides distended with blood, also the right ventricle; the coronary veins much distended on both sides; heart itself very large. *Abdomen.*—Liver large, full of blood; general engorgement of stomach and intestines. Stomach filled with undigested meat; internal surface engorged to near the pylorus. Spleen and pancreas healthy.

The stomach and its contents were boiled and the decoction considerably evaporated and handed over to Mr. Elliott, Professor of Chemistry in Georgetown College for analysis. This decoction was tested with peroxide of lead, as proposed by Marchand, and to Prof. Elliott and some others present appeared to give the blue color indicating the presence of strychnia. I did not perceive it. Several young gentlemen, who did not know what they were tasting, thought they could identify the same taste in the decoction and a very dilute solution of strychnia. I could scarcely say that I perceived any taste in either, but there was an impression made upon my fauces which I should consider the result of an exceedingly faint sensation of acrimony, rather than a taste very much alike. Further analysis was suspended for the present, with the intent that the decoction should be evaporated to dryness, and again tested by the peroxide of lead.

About this time it was considered desirable by the parties most interested, that the analysis should not for the present be completed, and shortly after Prof. Elliott resigned his situation in the college, and has been very much absent. He informed me, however, that he had satisfied himself, by evaporating a portion of the decoction and testing it with peroxide of lead, that strychnia was present.

Without further comment I submit these cases to the reflection of those who may deem them worthy of consideration.

May 15, 1849.

